# Regression of a venous malformation during angiotensin-converting enzyme inhibitor treatment for hypertension

**DOI:** 10.1016/j.jvscit.2022.09.004

**Published:** 2022-09-17

**Authors:** Sigurd Berger, Therese Halvorsen Bjark, Karsten Midtvedt, Rune Andersen

**Affiliations:** a Department of Radiology and Nuclear Medicine, Oslo University Hospital and Institute of Clinical Medicine, University of Oslo, Oslo, Norway; b Department of Plastic and Reconstructive Surgery, Oslo University Hospital, Oslo, Norway; c Department of Transplantation Medicine, Oslo University Hospital-Rikshospitalet, Oslo, Norway; d Department of Radiology and Nuclear Medicine, Oslo University Hospital, Oslo, Norway

**Keywords:** Therapeutics, Vascular malformations

## Abstract

Recent studies have reported that components of the renin-angiotensin system (RAS) are expressed in venous malformations by embryonic stem cell-like subpopulations. It has been hypothesized that these cells are sustained by the RAS and, therefore, could be a novel therapeutic target, using medications such as angiotensin-converting enzyme inhibitors. A young man with a symptomatic intramuscular venous malformation of the upper limb, and hypertension was treated with an angiotensin-converting enzyme inhibitor. After 8 months of treatment, we registered a considerable volume reduction of the venous malformation and a reduction in pain. Our observation warrants further research on the link between the RAS and venous malformations.

The overall incidence of congenital vascular malformations in the general population has been estimated to be 1.2%, with approximately two thirds of venous predominance.^1^^,^^2^ Venous malformations are lesions that can cause pain, swelling, functional impairment, and reduced quality of life.^3^^,^^4^ Traditionally, patients have been treated with surgery or sclerotherapy. However, the treatments have often been unsuccessful, especially for extensive malformations for which the currently available invasive treatment methods might not be an option at all. For these patients, several studies have shown promising effect of pharmacologic treatment with sirolimus, an inhibitor of the mammalian target of rapamycin (mTOR).^5^^,^^6^ However, owing to the unwanted side effects, the need for therapeutic drug monitoring, and cost issues, other treatment options are needed.

Recent studies have reported that embryonic stem cell-like subpopulations in venous malformations express components of the renin-angiotensin system (RAS).^7^, ^8^, ^9^ The authors of these studies have hypothesized that such primitive cells could be a novel therapeutic target by manipulation of the RAS using angiotensin-converting enzyme (ACE) inhibitors. In the present report, we have described an interesting observation that supports the hypothesis that ACE inhibitors could have the potential to reduce the volume and pain in venous malformations. The patient provided written informed consent for the report of his case details and imaging studies.

## Case report

**Fig 1:**
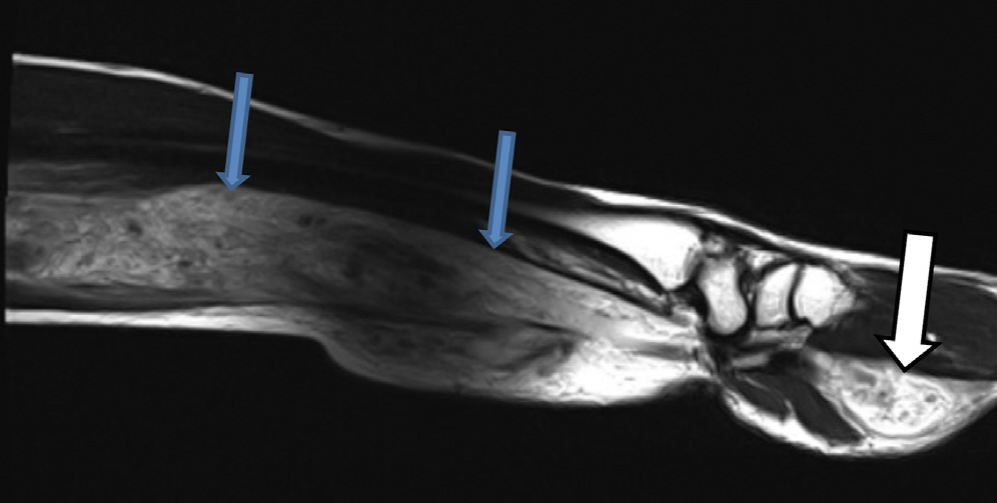
Sagittal T2-weighted magnetic resonance image showing extent of venous malformation of the left forearm (*blue arrows*) and hand (*white arrow*).

A 41-year-old man had, since childhood, experienced weakness of his left forearm and hand and activity-related pain. At 35 years of age, he had had a left-sided wrist fracture. During the next 2 years, the pain had worsened, with frequent episodes of swelling of the forearm. Because of his increased symptoms, magnetic resonance imaging (MRI) had been performed at the local hospital and showed an extensive, intramuscular lesion that extended from the elbow to the hand through the carpal tunnel (Fig 1). The lesion contained both phleboliths and large veins. He was referred to the national vascular anomaly center for a diagnostic evaluation and treatment. After discussion by the multidisciplinary team, a venous malformation was suspected and confirmed with ultrasound. The ultrasound scan showed compressible vascular structures and no sign of arteriovenous shunting. The patient had considerable symptoms, and different invasive treatment approaches were discussed.

**Fig 2:**
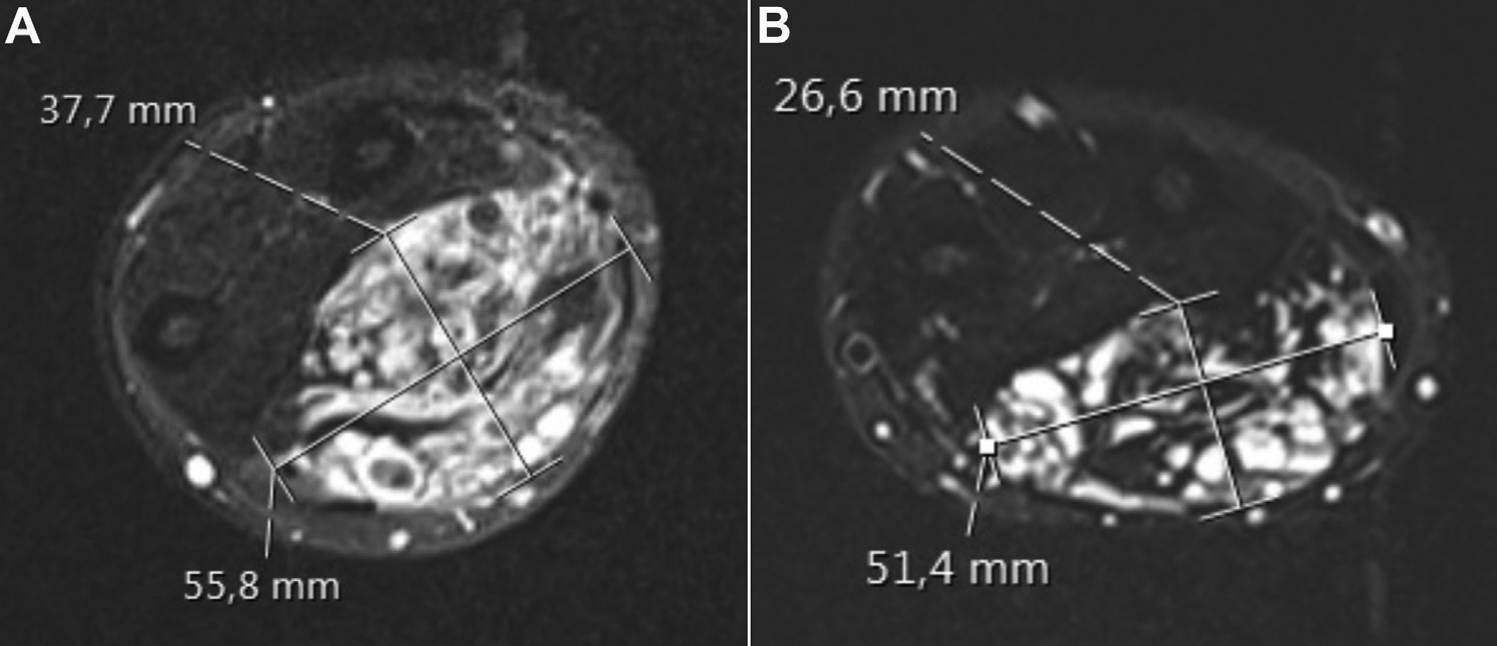
Axial short-T1 inversion recovery images of the left forearm before initiation of treatment with an angiotensin-converting enzyme (ACE) inhibitor **(A)** and after 8 months of ACE inhibitor treatment **(B)**. At the level of the forearm, the malformation had decreased from 56 × 38 mm to 51 × 27 mm.

Before any treatment of the venous malformation was initiated, he was diagnosed with hypertension after a routine clinical examination had revealed a blood pressure of 175/105 mm Hg. This was confirmed with 24-hour blood pressure monitoring. A routine workup, including blood tests, electrocardiography, and urinalysis revealed no discrepancies. At 5 months after the initial MRI, he started antihypertensive treatment with the ACE inhibitor enalapril (10 mg once daily). After initiation of the antihypertensive treatment, the patient reported rapid symptomatic improvement with less pain and swelling of his left forearm. A new MRI was performed after 8 months of ACE inhibitor treatment, which showed a significant size reduction of the malformation (Fig 2). To estimate the volume change, we added the area of the malformation on multiple adjacent MRI slices and then multiplied the sum by the slice thickness. The calculated volume of the malformation had decreased from 254.9 cm^3^ to 132.5 cm^3^, a reduction of 48.0%. Because of his significant symptomatic improvement, invasive treatment was not performed. The patient was followed up in the outpatient clinic during the fall of 2021. He was still taking enalapril as antihypertensive medication with no side effects and reported only mild, sporadic left forearm pain, probably due to thrombophlebitis episodes. A compression garment was provided, and he was advised to initiate treatment with a platelet inhibitor (enteric-coated aspirin [Albyl-E], 75 mg once daily). At a consultation 7 months later, he stated he was satisfied with his situation.

## Discussion

In the present case report, we have described a young man with a symptomatic venous malformation and hypertension treated with an ACE inhibitor. After 8 months of treatment with the ACE inhibitor, we had found a considerable volume reduction of the malformation both clinically and on MRI. Concurrently, the patient had reported significant pain reduction. Recent studies have implied a potential link between the RAS and venous malformations. Although we could not prove a cause-and-effect relationship between our patient’s ACE inhibitor treatment and his symptomatic improvement, we believe that our findings add relevance to this discussion.

Two years before his initial imaging study, the patient had had a wrist fracture, which might have been a contributing factor to his symptomatic progression. Although spontaneous regression of venous malformations is rare,^10^^,^^11^ a possibility exists that the observed clinical and radiologic regression were simply the natural history of the malformation, occurring coincidentally with the initiation of ACE inhibitor treatment. His symptoms could also have been caused by thrombophlebitis, causing stasis and swelling, which resolved spontaneously. His initial MRI had shown some phleboliths in the malformation; however, the imaging findings were inconclusive regarding whether the patient had had thrombophlebitis.

In vascular malformations, several somatic gene mutations leading to activation of the signaling pathways involved in vascular proliferation and angiogenesis have been identified. An activating mutation of angiopoietin-1 receptor (TIE-2), an endothelial receptor tyrosine kinase, and subsequent activation of the phosphatidylinositol 3-kinase (PI3K)/protein kinase B (AKT)/mTOR pathway, is believed to be a major cause of the development of sporadic venous malformations. Furthermore, in patients lacking TIE-2 mutations, activating mutations have been identified in PIK3CA (phosphatidylinositol-4,5-bisphosphonat 3 kinase catalytic subunit-alpha), an important part of the PI3K complex in the PI3K/AKT/mTOR pathway.^12^^,^^13^ The different molecular components of the PI3K/AKT/mTOR pathway could be targets for pharmacologic treatment. It has been recognized that sirolimus has the potential to reduce pain and improve the quality of life of patients with extensive venous (and lymphatic) malformations.^5^^,^^6^ However, the effect and potential unwanted side effects of sirolimus in the long term remain unknown, and further research on novel targeted therapies is warranted.

RAS is an endocrine system involved in the regulation of blood pressure, fluid and electrolyte balance, and systemic vasculature resistance. The physiologically active component of RAS is angiotensin II, through its interaction with angiotensin receptors (ATRs) 1 and 2. It is believed that ATR1 has proangiogenic effects, mainly through its interaction with vascular endothelial growth factor and the PI3K/AKT/mTOR pathway in endothelial cells.^14^ Recent studies have shown that the components of the RAS are expressed in both intramuscular and subcutaneous venous malformations, including prorenin receptor, ACE, ATR1, and ATR2.^7^ These components are expressed by embryonic stem cell-like subpopulations, both within and outside the endothelium of venous malformations.^8^^,^^9^ It has been hypothesized that these primitive cells give rise to cells in venous malformations and that the RAS might sustain these cells. Although a study has shown that RAS might activate the PI3K/AKT/mTOR pathway in other cell types,^15^ it is not yet known whether this interaction also exists in primitive cells of venous malformations and whether this possible interaction plays a major pathophysiologic role in such lesions.

We have not had experience with other patients with vascular malformations treated with ACE inhibitors, and the observation with the present patient has not changed our approach to the treatment of venous malformations. However, we intend to study further whether ACE inhibitors might have a therapeutic effect in these patients.

## Conclusions

Our observation of the clinical regression of a venous malformation after antihypertensive treatment with an ACE inhibitor warrants further research on the link between the RAS and primitive cells in venous malformations. Whether ACE inhibitors might have a role in the treatment of patients with venous malformations, either as monotherapy or combined with other drugs such as sirolimus, requires further exploration.
